# Neuromuscular Electrical Stimulation in Brachial Plexus Birth Injury Rehabilitation: A Systematic Review

**DOI:** 10.3390/medicina62061143

**Published:** 2026-06-11

**Authors:** Barış Celbek, Zeynep Hoşbay, Eda Urhun Keleş, Hayri Ömer Berköz, Adnan Yüksel

**Affiliations:** 1 Department of Physiotherapy and Rehabilitation, Institute of Graduate Studies, Biruni University, 34015 Istanbul, Türkiye; eda.urhun@yeditepe.edu.tr; 2 Physiotherapy Program, Department of Medical Services and Techniques, Vocational School of Health Services, Istanbul Aydin University, 34295 Istanbul, Türkiye; 3 Department of Physiotherapy and Rehabilitation, Faculty of Health Sciences, Biruni University, 34015 İstanbul, Türkiye; zhosbay@biruni.edu.tr; 4 Department of Physiotherapy and Rehabilitation, Faculty of Health Sciences, Yeditepe University, 34755 İstanbul, Türkiye; 5 Department of Plastic Reconstructive and Aesthetic Surgery, İstanbul Faculty of Medicine, Istanbul University, 34093 İstanbul, Türkiye; 6 Department of Child Health and Diseases, Faculty of Medicine, Biruni University, 34015 İstanbul, Türkiye

**Keywords:** brachial plexus injuries, neuromuscular electrical stimulation, rehabilitation, pediatric physiotherapy, muscle strength, range of motion

## Abstract

*Background and Objectives*: Brachial plexus birth injury (BPBI) is a peripheral nerve injury occurring during birth that may result in upper-extremity weakness and functional impairment. This systematic review aimed to evaluate the effects of neuromuscular electrical stimulation (NMES) on motor function, muscle strength, range of motion, and upper-extremity function in children with BPBI. *Materials and Methods*: This systematic review was conducted according to PRISMA guidelines and registered in PROSPERO. PubMed, CINAHL, Scopus, Web of Science, PEDro, and the Cochrane Library were searched from inception to 5 May 2026. Only randomized controlled trials were included. Methodological quality was assessed using the PEDro scale, and risk of bias was evaluated using the RoB 2 tool. *Results*: Seven randomized controlled trials involving 197 participants were included. Several studies reported improvements in shoulder abduction, elbow flexion, wrist extension, muscle strength, and motor function following NMES compared with conventional therapy. The combination of NMES and constraint-induced movement therapy demonstrated favorable outcomes in functional performance. However, substantial heterogeneity was observed across studies regarding participant characteristics, NMES parameters, treatment duration, and outcome measures. The certainty of evidence ranged from low to very low. *Conclusions*: Current evidence suggests that NMES may serve as a potential adjunct to conventional rehabilitation in children with BPBI. However, given the low to very low certainty of the evidence, high risk of bias, and substantial clinical and methodological heterogeneity among the included studies, definitive clinical recommendations cannot currently be made. Future well-designed randomized controlled trials using standardized protocols, consistent outcome measures, and longer follow-up periods are warranted.

## 1. Introduction

Brachial plexus birth injury (BPBI), also known as neonatal brachial plexus palsy (NBPP), is defined as weakness or flaccid paralysis of the upper extremity diagnosed soon after birth, resulting from injury to one or more cervical and thoracic nerve roots (C5–T1) due to traction or compression of the brachial plexus during delivery [1]. BPBI encompasses a spectrum of clinical presentations depending on the extent and location of nerve involvement. Erb’s palsy represents the most common upper-trunk subtype of BPBI and primarily involves the C5–C6 nerve roots. According to the Narakas classification, brachial plexus birth injury (BPBI) is clinically categorized into four groups based on the extent of nerve root involvement. These include Erb’s palsy (C5–C6), extended upper trunk involvement (C5–C7), which may present with or without early wrist extension, and total plexus paralysis (C5–T1), which may be accompanied by Horner syndrome [2]. BPBI may be accompanied by a range of clinical manifestations depending on the extent of nerve involvement. These include muscle weakness, soft tissue contractures, progressive glenohumeral joint deformity and/or instability, and scapular dyskinesia. As a peripheral nerve injury, BPBI may also present with sensory and autonomic dysfunction in addition to motor impairment. Secondary complications include joint contractures, muscle shortening, skeletal deformities, limb length discrepancies, abnormal posture, and impaired motor coordination, which may adversely affect upper-extremity use and motor development [3]. Despite improvements in obstetric care, BPBI remains one of the most common birth-related peripheral nerve injuries. Recent evidence indicates that the pooled overall incidence of BPBI is 1.74 per 1000 live births, although reported rates vary from 0.38 to 5.1 per 1000 live births across different populations. Furthermore, a large population-based cohort study from California reported an incidence of 1.23 per 1000 live births, while a recent study from Türkiye reported an incidence of 0.9 per 1000 live births [4,5,6]. The type and location of nerve damage determine the severity of the injury, ranging from moderate neuropraxia to more serious conditions such as root avulsion [2]. While many infants recover on their own within the first few months of life, many struggle with persistent weakness, muscular imbalance, and joint contractures that can limit their ability to use their extremities functionally [7].

To minimize additional challenges and promote motor recovery, early and comprehensive physiotherapy is considered crucial in the rehabilitation of newborns with BPBI [8]. Traditional rehabilitation treatments usually focus on sensory stimulation, functional training, and passive and active range-of-motion exercises [9,10]. To improve motor function and promote neuromuscular activation, additional therapeutic approaches may be necessary if recovery is incomplete [11]. Neuromuscular Electrical Stimulation (NMES) is one such intervention that strengthens weak muscles and promotes motor learning by delivering electrical impulses to peripheral nerves, thereby eliciting muscle contractions [12].

NMES has been widely used to improve functional outcomes, reduce atrophy, and facilitate muscle re-education in a variety of pediatric and neurological diseases. By giving the afflicted muscles repeated sensory-motor input, NMES may promote neuronal plasticity and hasten reinnervation in the context of BPBI [12]. However, there is still conflicting and scant data on its effectiveness in babies and kids with BPBI [11]. While some research has shown that NMES treatment improves muscle strength, joint mobility, and functional performance, other studies have revealed little to no additional effect when compared to conventional therapy alone [13,14].

Highly varied stimulation settings, such as variations in frequency, pulse duration, duty cycle, electrode placement, and treatment duration, have been used in prior research on children with BPBI [10,15,16,17]. Potential increases in muscle strength, active range of motion (AROM), and general upper limb function have been reported, especially in muscles like the deltoid and biceps, which are frequently impacted in BPBI [16,17,18]. However, current information is largely limited to small case studies and variable approaches, and there is still no defined protocol for the optimal NMES parameters or treatment duration in this population [19,20,21].

A comprehensive examination of the current research is necessary, considering the increased interest in further electrical stimulation approaches and the therapeutic significance of maximizing motor recovery in BPBI. This systematic review aimed to evaluate the effects of NMES on motor function, muscle strength, range of motion, and upper-extremity function in children with BPBI, and to summarize the NMES protocols, outcome measures, and the current evidence available in the literature.

## 2. Materials and Methods

The design and methods for this systematic review were performed according to the guidelines in the Cochrane Handbook for Systematic Reviews of Interventions (Version 6.0) and reported in line with Preferred Reporting Items for Systematic Reviews and Meta-Analyses 2020 (PRISMA 2020, Supplementary Material File S1). These systematic review protocols have been registered prospectively at the National Institute of Health Research PROSPERO (CRD420261332718).

### 2.1. Search Strategy

A comprehensive literature search was independently conducted in May 2026 across six electronic databases (PubMed, CINAHL, Scopus, Web of Science, PEDro, and the Cochrane Library), as well as relevant grey literature sources, and was supplemented by manual screening of the reference lists of all included studies and relevant review articles. This process was carried out independently by two reviewers (BC, EUK) to minimize bias. All databases were searched from their inception to 5 May 2026. The search strategy was developed and refined in consultation with a librarian and content experts, combining controlled vocabulary (Medical Subject Headings [MeSH]) and free-text terms related to “brachial plexus” and “neuromuscular electrical stimulation.” The strategy was first tested and calibrated in PubMed following the Peer Review of Electronic Search Strategies (PRESS) guidelines [22]. Database-specific search strings are presented in Supplementary Material File S2. No restrictions were applied regarding publication date or geographical location, but only studies published in English or Turkish were considered.

All retrieved records were imported into Rayyan (a free web-based systematic review tool; Qatar Computing Research Institute, Qatar; https://www.rayyan.ai/, accessed on 5 May 2026) for screening [23]. Duplicate records were automatically identified and removed within Rayyan before the title and abstract screening process.

### 2.2. Eligibility Criteria

The Population, Intervention/Exposure, Comparison, Outcome, and Study design (PICOS) framework was used to define the eligibility criteria.

The Inclusion criteria for the studies were:

1. Population: Infants and children diagnosed with brachial plexus birth injury (BPBI), including Erb’s palsy and other BPBI subtypes when reported.
2. Intervention/Exposure: Neuromuscular electrical stimulation (NMES), either as a standalone treatment or combined with conventional physiotherapy.
3. Comparison: Conventional physiotherapy, other rehabilitation interventions, placebo, or no intervention.
4. Outcome: Studies reporting at least one of the following: functional recovery, muscle strength, range of motion, motor performance.
5. Study design: Randomized controlled trials (RCTs)

The Exclusion criteria for the studies were:

1. Studies not written in English or Turkish were excluded due to language restrictions.
2. Studies with no other exercise intervention, placebo, or control groups.
3. Studies that reported no relevant outcome measures.
4. Observational studies, in vitro studies, in silico studies, animal studies, quasi-experimental studies, case reports, and case series were excluded.

All included studies involved infants and children diagnosed with Erb’s palsy. Participant age ranged widely from the neonatal period (approximately 2–53 days) to early childhood (up to 8 years), indicating substantial clinical variability across studies. Information regarding the severity of brachial plexus injury and timing of intervention initiation post-birth was extracted when reported; however, these variables were inconsistently available across studies.

### 2.3. Data Extraction

Study selection was performed by screening the titles and abstracts of retrieved records according to predefined eligibility criteria. In cases where eligibility could not be determined from the abstract, full-text articles were assessed. Studies meeting the predefined eligibility criteria were included after independent evaluation by both reviewers, with any disagreements resolved through discussion. The reviewers met and discussed the articles by title and abstract to determine inclusions. Any disagreements were resolved by a third reviewer (ZH). After that, the same reviewers assessed the full texts of the remaining studies against the inclusion and exclusion criteria. The same researchers met and discussed each paper to determine inclusion, and any disagreements were resolved by the third reviewer. Final decisions for the studies were then made.

The following data from the included studies were extracted: the title of the study, the authors, year of publication, descriptive statistics of the groups (age, gender, disease type, number of participants), inclusion criteria, outcome measures, intervention details (intensity, duration, and frequency), and comparisons with control/placebo groups.

### 2.4. Assessment of Methodological Quality

The methodological quality of the included studies was independently assessed (BC, EUK) using the PEDro scale, which has 11 criteria for assessing the external and mainly internal validity of randomized controlled studies, from which points are scored from criterion 2 to 11, resulting in a maximum score of 10. Disagreements between reviewers were resolved by discussion with a third researcher (ZH). PEDro scores of 0–3 are considered ‘poor’, 4–5 ‘fair’, 6–8 ‘good’, and 9–10 ‘excellent’ [24,25].

### 2.5. Risk-of-Bias Assessment

Using the Cochrane Risk of Bias tools 2.0 (ROB 2.0) to evaluate the risk of bias in randomized controlled trials, the methodological quality of each included study was assessed [26]. A third researcher was consulted to settle any discrepancies in the experts’ evaluations. The randomization procedure, handling of missing outcome data, outcome assessment, and selection of reported results were all evaluated as aspects of the research’s methodological quality. Based on the ROB 2.0 criteria, these domains were classified as low risk, high risk, or some concerns. The risk-of-bias assessment tool is used to format traffic light plots of the domain-level assessments for each outcome and weighted bar plots showing the distribution of risk-of-bias judgments within each bias domain.

### 2.6. Data Synthesis

Due to significant clinical and methodological heterogeneity among the included studies, a meta-analysis was not performed. Studies differed substantially in NMES protocols (frequency, pulse duration, current type, target muscle group, and treatment duration), participant age ranges, intervention types, and outcome measures. Most outcomes were reported by only one or two studies, and a considerable proportion provided only *p*-values without means or standard deviations, precluding calculation of standardized effect sizes or I^2^ heterogeneity analysis. Consequently, a narrative synthesis approach was adopted in accordance with the PRISMA guidelines and the Synthesis Without Meta-Analysis (SWiM) reporting guideline [27]. Extracted data were organized into structured summary tables covering participant characteristics, intervention protocols, outcome measures, and key findings (Table 1 and Table 2). Findings were then grouped by outcome domain range of motion, muscle strength and motor function, functional performance and the direction of effect was assessed for each domain across studies. Cross-study comparisons were limited to studies sharing comparable outcome instruments. The overall body of evidence within each domain was subsequently appraised in light of methodological quality (PEDro scores), risk of bias (RoB 2.0), and the clinical comparability of included populations. The certainty of evidence for each outcome was assessed using the Grading of Recommendations Assessment, Development and Evaluation (GRADE) approach [28]. As a meta-analysis could not be performed, GRADE was applied to the narrative synthesis in accordance with published guidance for systematic reviews without pooled estimates. Starting from high certainty (all included studies were RCTs), the evidence was downgraded based on five domains: risk of bias, inconsistency, indirectness, imprecision, and publication bias. The overall certainty was classified as high, moderate, low, or very low. Results are presented in Table 3.

**Table 1:** Characteristics of the studies and participants.

Study	Participant/Sample	Intervention	Outcome Measures	Main Results
Okafor et al. (2008), Nigeria [10]	*n* = 16 infants with Erb’s palsy. Age from 2 to 52 days, both sexes. Experimental: *n* = 8 Age (days) mean: 22 CT: *n* = 8 Age (days) mean: 22.3	Experimental: Electrical stimulation was applied for 15 min to each of the three muscle groups (shoulder abductors, elbow flexors, and wrist extensors) for a total of 45 min, three times a week for six weeks. CT: applying an aeroplane splint to the afflicted upper limb after passive mobilization and soft tissue manipulation (massage). Program for therapy: 45 min, three times a week for six weeks.	The Universal Goniometer measures shoulder abduction, elbow flexion, wrist extension, and active range of motion. The tape rule measures the circumference of the mid-arm.	Body structure and function: ROM (mean) before and after treatment: Experimental: 8.75/36.9 for shoulder abduction, 10.6/35 for elbow flexion, and 9.38/29.4 for wrist extension. CT: 6.88/20 for shoulder abduction; 10/24.4 for elbow flexion; 8.75/24.4 for wrist extension; Mid-arm circumference before and after treatment (mean): CT arm circumference: 14.25/15.69 cm; experimental arm circumference: 14.63/16.56 cm Participation/Activity: not rated All measures showed significant changes, with the experimental group showing a greater increase than the CT group. For early restoration of upper limb function in patients with Erb’s palsy, electrical stimulation is more effective than CT.
Sherief, A. A. A. (2011), Egypt [29]	*n* = 30 children with Erb’s palsy. Age from 1 to 5 months, both sexes Experimental: *n* = 15. Control: *n* = 15.	Experimental: Physiotherapy program in addition to electrical stimulation applied over the deltoid and forearm muscles. Electrical stimulation was used during the exercise sessions (1.5 h per session, 3 sessions per week for 3 months). Control: The same physiotherapy program was applied with the use of a static splint.	Electroneurography (ENoG): Percentage of degeneration of the deltoid and biceps brachii muscles using a computerized EMG device. Toronto Active Motion Scale (TAMS): Motor functional assessment (Shoulder flexors, extensors, abductors, external rotators, and Elbow flexors).	Body structure and function: Percentage of Degeneration (mean ± SD): Deltoid degeneration (mean ± SD): Experimental 68.8 → 41.9; Control 71.4 → 20.5 (*p* = 0.001) Biceps degeneration: Experimental 69.6 → 53.2; Control 69.8 → 21.6 (*p* = 0.001) TAMS (mean ± SD): Shoulder flexion 2.76 → 4.58 vs. 2.62 → 5.43 (*p* = 0.001); Abduction 3.43 → 5.6 vs. 3.27 → 6.55 (*p* = 0.001); External rotation 2.22 → 5.2 vs. 2.55 → 6.77 (*p* = 0.001). Participation: not rated
Elnaggar et al. (2016), Saudi Arabia [17]	*n* = 42 children with Erb’s palsy. Age from 3 to 5 years, both sexes. Experimental: *n* = 21. Age (years) mean (SD): 3.67 (0.73) Control: *n* = 21. Age (years) mean (SD): 4.05 (0.80)	Experimental: weight-bearing exercises combined with a physiotherapy treatment and NMES. For a total of three months, an alternating symmetrical biphasic current was applied for 40 min each day. Control: a physiotherapy treatment that focuses on functional performance and loading on the afflicted arm (40 min per day for three consecutive months).	Norland XR-46 dual-energy x-ray absorptiometry: BMD Mallet Score Test: (Abduction, External Hand to mouth, hand to neck, hand to spine, and rotation.)	Body structure and function: Affected humerus’s mean total BMD (pre/post): Control Group (0.354/0.364); Experimental Group (0.363/0.387) MSS scores (mean) before and after treatment: Experimental Group: External Rotation (3.33/3.38); Abduction (2.95/3.43). Control Group: External Rotation (2.48/2.95); Abduction (2.62/2.90). Activity: Mean MSS scores before and after treatment: Experimental Group: hand to mouth (2.57/3.43), hand behind head (2.33/3.71), and hand to back (2.81/3.57). Hand behind head (2.62/3.14), hand to back (2.48/3.10), and hand to mouth (2.24/2.81) comprise the control group. Participation: not rated. BMD means and all Mallet scale components favor the experimental group. For children with Erb’s palsy, NMES during weight-bearing exercises is an efficient and straightforward method to enhance shoulder function and BMD.
Abdelaziz et al. (2022), Egypt [30]	*n* = 30 children with Erb’s palsy. Age from 1 to 3 years, both sexes Experimental: *n* = 15. Control: *n* = 15.	Experimental: Physiotherapy program in addition to reciprocal electrical stimulation for biceps and triceps muscles. Electrical stimulation was used during the exercise sessions (3 sessions per week for 3 months). Control: The same physiotherapy program was applied.	Electroneurography (ENoG): Percentage of degeneration of the biceps brachii muscles using a computerized EMG device.	Body structure and function: Percentage of Degeneration (mean ± SD): Biceps degeneration: Control (Group A): Pre 69.21 ± 2.32 → Post 59.98 ± 2.5 Experimental (Group B): Pre 68.75 ± 3.94 → Post 38.33 ± 8.23 (*p* = 0.0001) Participation: not rated
Justice et al. (2023), USA [31]	*n* = 17 neonatal brachial plexus palsy. Age between 3 and 9 months, both sexes. Experimental: *n* = 10 Age (months) mean (SD): 4 ± 2 Sham: *n* = 7 Age (months) mean (SD): 4 ± 1	Experimental: Home-based NMES applied to the biceps muscle using a preprogrammed Empi Continuum™ device (10 s on, 30 s off, 35 Hz, 300 μs, Level 4). Parents administered NMES daily for 30 min during playtime for 3 months. Control/Sham: Same device with sham settings (0 s on, 60 s off, 35 Hz, 48 μs, Level 4) applied under identical conditions for 3 months.	Active range of motion (AROM) for shoulder and elbow movements (flexion, abduction, extension, external rotation, pronation, supination), biceps muscle strength (MRC scale 0–5), and bilateral limb morphometric measurements were assessed at baseline and at 1-, 2-, and 3-month follow-ups	Body Structure and Function: Elbow flexion AROM: Significant improvement in NMES group after 1 month (31° vs. –3°, *p* = 0.047). No other significant differences in shoulder, elbow, forearm AROM, or biceps strength at 2–3 months. Muscle strength: Similar between groups at baseline; no significant changes observed beyond elbow flexion. Morphometrics: No significant differences in limb length or girth between groups over 3 months. Participation: Home NMES usage was similar between groups. Treatment group participants completed more sessions in the first month (24/30) compared with the control/sham group (18/30).
Elnegamy, T. E. (2024), Saudi Arabia [32]	*n* = 40 children with Erb’s palsy. Age between 2 and 5 months, both sexes Experimental: *n* = 20 Age (months) mean (SD): 2.99 ± (0.90) Control: *n* = 20 Age (months) mean (SD): 2.87 ± (0.71)	Experimental: Standard physical therapy plus reciprocal electrical stimulation (RES) applied to elbow flexors and extensors for 15 min, three sessions per week for 12 weeks. Control: Received standard physical therapy	Electroneurography (ENoG): Percentage of degeneration of the triceps and biceps brachii muscles using a computerized EMG device. Toronto Active Motion Scale (TAMS): Motor functional assessment of triceps and biceps brachii muscles.	Body structure and function: Percentage of Degeneration (mean ± SD): No significant pre-treatment difference in RD of biceps and triceps between control and experimental groups (*p* > 0.05). Post-treatment values significantly improved in the experimental group for both muscles (*p* < 0.001). Biceps—Control: Pre 64.47 ± 4.19 → Post 57.84 ± 3.8 Experimental: Pre 63.35 ± 3.49 → Post 49.84 ± 6.87 (*p* < 0.001). Triceps—Control: Pre 62.37 ± 3.10 → Post 56.84 ± 4.56. Experimental: Pre 61.31 ± 4.41 → Post 48.79 ± 2.10 (*p* < 0.001). Activity (TAMS): No baseline difference between groups (*p* > 0.05). Post-treatment scores significantly increased in the experimental group for both biceps and triceps (*p* < 0.001). Biceps—Control: 2 → 3; Experimental: 2 → 4 (*p* < 0.001). Triceps—Control: 2 → 3; Experimental: 2 → 4 (*p* < 0.001). Participation: not rated
Tariq et al. (2024), Pakistan [33]	*n* = 22 children with Erb’s palsy. Age between 6 and 8 years, both sexes. Experimental: *n* = 11. Age (years) mean (SD): 4.3 ± 1.2 NMES Group: *n* = 11. Age (years) mean (SD): 4.5 ± 1.6	Experimental: Constraint-Induced Movement Therapy (CIMT) for 3 weeks, ≥6 h/day, combined with NMES for 6 weeks, 4 sessions/week. NMES Group: NMES alone for 6 weeks, 4 sessions/week.	Mallet Score Test (MST) Box and Block Test	Body Structure and Function: Mallet score: Significant improvement within the CIMT + NMES group over time (1.00 ± 0.00 to 4.06 ± 0.70, *p* < 0.001). Box and Block test: Marked increase from 9.64 ± 2.25 to 130.55 ± 11.06 after 6 weeks (*p* < 0.001). Between-group comparison: Greater, though not statistically significant, improvement in CIMT + NMES group compared to NMES-only group (Mallet *p* = 0.101; Box and Block *p* = 0.054). Activity and Participation: Children receiving CIMT + NMES demonstrated superior improvement in upper limb coordination and dexterity, reflected by greater Box and Block test gains and enhanced task-oriented functional performance. Overall Effect: CIMT combined with NMES resulted in greater functional recovery, muscle recruitment, and coordination than NMES alone, suggesting a synergistic effect in promoting motor function in children with Erb’s palsy. Participation: not rated

*n*: number of participants; CT: Conventional Therapy; NMES: Neuromuscular Electrical Stimulation; RES: Reciprocal Electrical Stimulation; AROM: Active Range of Motion; ROM: Range of Motion; BMD: Bone Mineral Density; TAMS: Toronto Active Motion Scale; ENoG: Electroneurography; SD: Standard Deviation; CIMT: Constraint-Induced Movement Therapy; Empi Continuum™: Brand name of NMES device (used in home-based NMES); Pre/post: before and after intervention.

**Table 2:** NMES intervention protocols.

Study	NMES Training Protocol	Training Program	Frequency	Current Characteristics	Intensity	Pulse Duration (μs)
Okafor et al. (2008) [10]	NMES was applied for 15 min to each of the three muscle groups (shoulder abductors, elbow flexors, and wrist extensors) for a total of 45 min, three times a week for six weeks.	6 wk × 3 sessions/wk	Not reported	Not reported	Not reported	Not reported
Sherief, A. A. A. (2011) [29]	Not reported any information about the protocol	12 wk × 3 sessions/wk	Not reported	Not reported	Not reported	Not reported
Elnaggar et al. (2016) [17]	Initially, 10 pps producing a tapping sensation; increased to 30 pps to produce muscle contraction; total duration 15 min; duty cycle initially 10 s on/20 s off, then changed to 15 s on/15 s off when tolerated	12 wk × 7 sessions/wk	Initially 10 pps, then increased to 30 pps	Pulsed current	Gradually and slowly increased to each child’s tolerance, only when current was on	300 μs
Abdelaziz et al. (2022) [30]	20 min per session, 10 s ramp-up and 10 s ramp-down applied alternately to both muscles	12 wk × 3 sessions/wk	30 Hz	Pulsed current	Maximal tolerated	1000 μs
Justice et al. (2023) [31]	Experimental: Home-based 30 min. NMES applied to biceps during play sessions Sham: Home-based NMES device with sham settings applied to biceps	12 wk × 7 sessions/wk	35 Hz	Experimental: Symmetrical waveform, simultaneous cycling, ramp time 2 s Sham: Same but ramp time 0s	Both groups reported Level 4	Experimental: 300 μs Sham: 48 μs
Elnegamy, T. E. (2024) [32]	15 min per session, patient in supine position with affected arm beside the body	12 wk × 3 sessions/wk	50 Hz	Rectangular pulse	Gradually increased from low to a level producing gentle, visible muscle contraction; maintained throughout the session	1000 μs
Tariq et al. (2024) [33]	Experimental: Electrical stimulation applied to wrist extensors, combined with constraint-induced movement therapy (CIMT) NMES Group: Electrical stimulation applied to wrist extensors	6 wk × 4 sessions/wk	Not reported	Not reported	Not reported	Not reported

pps: pulses per second; Hz: Hertz (frequency of stimulation); μs: microseconds (pulse duration); s: seconds; Ramp-up/Ramp-down: Gradual increase/decrease in current intensity; Level 4: Intensity setting on the NMES device (device-specific); Sham: Placebo NMES or inactive stimulation; Rectangular pulse/Pulsed current/Symmetrical waveform: Types of electrical current waveform.

**Table 3:** GRADE Evidence Profile of Included Outcomes.

Outcome	No. of Studies (Participants)	Certainty of the Evidence (GRADE)	Summary of Findings
Range of Motion (ROM)	2 studies (*n* = 33)	VERY LOW ^a^^,^^b^^,^^c^	NMES may improve shoulder abduction, elbow flexion, and wrist extension compared to conventional therapy alone, but the evidence is very uncertain.
Motor Function (TAMS)	2 studies (*n* = 70)	LOW ^a,c^	NMES may improve motor function as assessed by the Toronto Active Motion Scale; evidence is limited by risk of bias and imprecision.
Shoulder Function (Mallet Score)	2 studies (*n* = 64)	VERY LOW ^a^^,^^b^^,^^c^	NMES may improve Mallet score components; however, the between-group difference was not statistically significant in one study (*p* = 0.101), and comparators differed substantially between studies.
Electrophysiological Outcomes (ENoG)	3 studies (*n* = 100)	VERY LOW ^a^^,^^b^^,^^d^	NMES may reduce the percentage of muscle fibre degeneration; however, ENoG is a surrogate outcome and its clinical relevance remains uncertain.
Functional Performance (Box and Block)	1 study (*n* = 22)	VERY LOW ^a,e^	The between-group difference was not statistically significant (*p* = 0.054). Evidence is derived from a single underpowered study; the independent effect of NMES cannot be isolated due to the concurrent CIMT application.

Footnotes: ^a^ Risk of Bias: Downgraded by one level. Five of seven included studies (Okafor et al., Sherief, Abdelaziz et al., Elnaggar et al. 2016, Tariq et al.) did not report assessor blinding or allocation concealment, resulting in a high overall risk of bias (RoB 2.0) [10,17,29,30,33]. Only one study (Justice et al.) was rated as low overall risk of bias [31]. ^b^ Inconsistency: Downgraded by one level. Substantial clinical heterogeneity was observed across studies in NMES target muscle groups (deltoid, biceps, triceps, wrist extensors), stimulation parameters (pulse duration: 300–1000 µs; frequency: 30–50 Hz), session duration, and total treatment duration. Although the direction of effect consistently favored NMES, effect magnitudes could not be compared across studies. ^c^ Imprecision: Downgraded by one level. Confidence intervals were not reported in most studies; the majority reported only *p*-values. Individual study sample sizes were small (range: *n* = 16–42), and the total number of participants for these outcomes was below the Optimal Information Size (OIS). Effect sizes could not be calculated. ^d^ Indirectness: Downgraded by one level. Electroneurography percentage of degeneration is a surrogate (electrophysiological) outcome. Its relationship to clinically meaningful functional recovery has not been directly demonstrated in the included studies, limiting the clinical interpretability of these findings. ^e^ Very Serious Imprecision: Downgraded by two levels. Evidence is derived from a single study with a very small sample size (*n* = 22). The between-group comparison did not reach statistical significance (Mallet: *p* = 0.101; Box and Block Test: *p* = 0.054). The study was likely underpowered, and the independent contribution of NMES cannot be determined due to concurrent CIMT application.

## 3. Results

### 3.1. Study Selection

**Figure 1:**
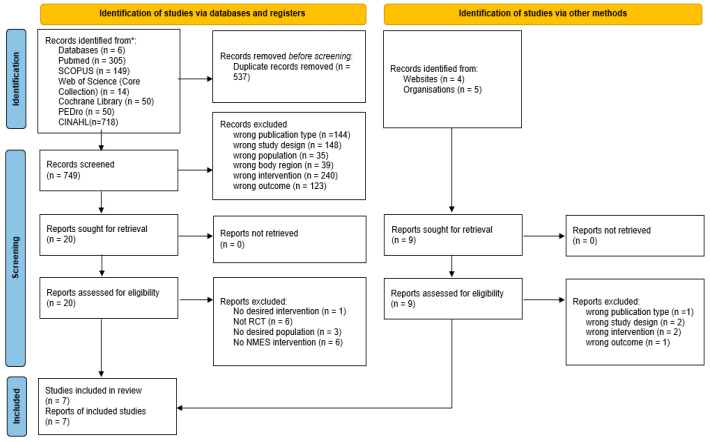
Flow chart. * Number of records identified from each database is reported separately rather than as a combined total, in accordance with the PRISMA 2020 flow diagram.

The study selection process is summarized in a flow chart (Figure 1). After removing duplicates, a total of 749 records were identified. After screening titles and abstracts, 729 studies were excluded. Full-text assessment of the remaining articles resulted in the inclusion of seven randomized controlled trials (RCTs) that met all eligibility criteria and were incorporated into the qualitative synthesis of this review [10,17,29,30,31,32,33]. Across these studies, a total of 197 participants were included, consisting of 103 infants and 94 children, including both boys and girls.

### 3.2. Methodological Quality Assessment

The PEDro scores of the included studies ranged from 5 to 8, with a mean score of 5.7. Four studies scored 5/10 [10,29,30,33], two studies scored 6/10 [17,32], and one study scored 8/10 [31]. Random allocation of participants was reported in all studies. Baseline comparability between study and control groups was ensured in all included studies. Between-group differences and point estimates were reported in all studies. Allocation concealment was reported in one study [17]. Participant blinding was reported in one study [31], while assessor blinding was reported in two studies [31,32]. Adequate follow-up outcomes were reported in all studies, and no study reported intention-to-treat analysis. Table 4 presents the PEDro scores and detailed methodological criteria for each included study.

### 3.3. Risk of Bias 2.0

**Figure 2:**
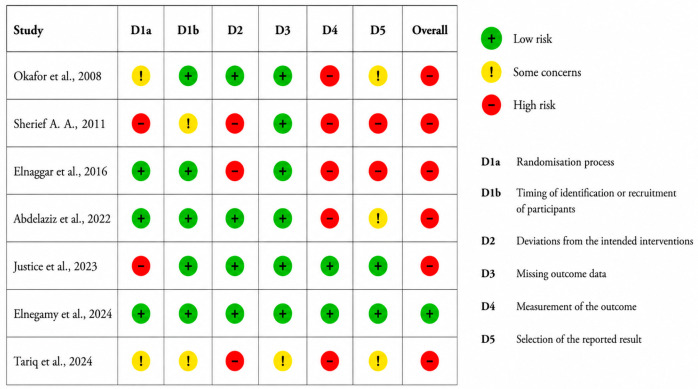
Risk of bias summary [10,17,29,30,31,32,33].

**Figure 3:**
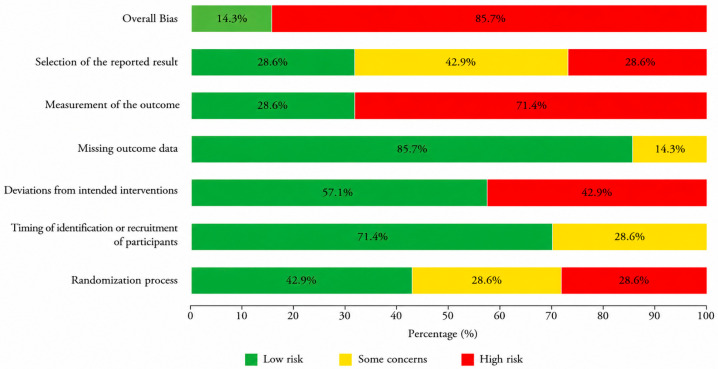
Risk of bias graph.

The risk of bias summary and visualization are reported in Figure 2 and Figure 3. Only one study (14.3%) was assessed as having a low overall risk of bias. Regarding the randomization process (Domain 1a), 42.9% of the studies were judged as low risk, while 28.6% presented some concerns, and 28.6% showed a high risk of bias. In the timing of identification or recruitment of participants (Domain 1b), most of the studies (71.4%) demonstrated a low risk of bias, whereas 28.6% were rated as having some concerns. With respect to deviations from intended interventions (Domain 2), 57.1% of the studies were classified as low risk, while 42.9% were considered to have a high risk of bias. For missing outcome data (Domain 3), a substantial proportion (85.7%) of the studies showed a low risk of bias, with the remaining 14.3% having some concerns. The measurement of outcomes (Domain 4) was identified as the most critical source of bias, with 71.4% of studies rated as high risk and only 28.6% as low risk. In the selection of the reported results (Domain 5), 42.9% of the studies demonstrated some concerns, while both low-risk and high-risk ratings were observed in 28.6% of the studies each. Overall, 85.7% of the studies were evaluated as having a high risk of bias, and only 14.3% were assessed as low risk across domains.

### 3.4. Qualitative Data Synthesis

#### 3.4.1. Outcomes

This review focused on the functional outcomes assessed in children with BPBI across the included studies. Upper limb active range of motion (AROM) was evaluated using a Universal Goniometer for shoulder abduction, elbow flexion, and wrist extension, while mid-arm circumference was measured with a tape rule in one study [10]. Bone mineral density (BMD) was assessed using dual-energy X-ray absorptiometry (DEXA) in another study [17]. Shoulder function, including abduction, external rotation, hand-to-neck, hand-to-spine, and hand-to-mouth movements, was measured using the Mallet Score System (MSS) [17]. Electroneurography (ENoG) was used to quantify the percentage of degeneration in the deltoid, biceps brachii, and triceps muscles [29,30,32]. Motor function of shoulder and elbow muscles was assessed with the Toronto Active Motion Scale (TAMS) in multiple studies [29,32]. Additional functional outcomes included active range of motion and biceps muscle strength measured by the MRC scale, along with bilateral limb morphometric measurements at baseline and follow-ups [31]. The Mallet Score Test and the Box and Block Test were used to assess fine and gross motor skills [33]. For each included research study, comprehensive intervention and outcome data are shown in Table 1 and Table 2.

#### 3.4.2. Interventions

The effects of different physiotherapy therapies, such as electrical stimulation and NMES, on upper limb function in children with BPBI were investigated in this systematic review. There were seven investigations in all, with various control and comparative groups. Passive mobilization, soft tissue manipulation, and the use of an airplane splint were contrasted with electrical stimulation administered to the wrist extensors, elbow flexors, and shoulder abductors [10]. Stressing loading on the afflicted arm and functional performance, physiotherapy in conjunction with NMES during weight-bearing activities was compared with physiotherapy alone [17]. Physiotherapy alone or in conjunction with a static splint was contrasted with electrical stimulation or reciprocal electrical stimulation [29,30]. Standard physical therapy combined with NMES was compared to standard physical therapy alone [32]. In another study, home-based NMES administered by parents was evaluated against a sham NMES protocol [31], while Constraint-Induced Movement Therapy in combination with NMES was compared with NMES alone [33].

The length, frequency, and severity of the interventions varied; they included organized sessions lasting 1.5 h, three times a week for three months, CIMT combined with NMES for six weeks, and daily home-based NMES for 30 min over three months. Electrical stimulation parameters, including session length, frequency, and targeted muscle groups, were tailored according to each study protocol. The characteristics of the included studies and detailed intervention protocols are presented in Table 3 and Table 1.

### 3.5. Study Characteristics

Seven randomized controlled trials (RCTs) [10,17,29,30,31,32,33] were included, with sample sizes ranging from 16 to 42 participants diagnosed with BPBI, aged 1 month to 8 years. Intervention durations varied from 6 weeks to 3 months, with treatment frequencies of three to four sessions per week. Applied physiotherapeutic approaches included NMES [17,29,30,31,32,33], static splinting [29], and conventional physiotherapy [10,29,30,32] and constraint-induced movement therapy (CIMT) combined with NMES [33].

Outcome measures comprised goniometric range of motion (ROM) [10,31], Toronto Active Motion Scale (TAMS) [29,32], Mallet Grading System [17,33], electromyography (EMG) [29,30,32], and dual-energy X-ray absorptiometry (DEXA) for bone mineral density (BMD) [17]. Standard physical treatment programs, sham stimulation protocols, or conventional physiotherapy were administered to comparison groups. Shoulder and elbow range of motion, muscle strength, motor function, and electrophysiological characteristics were all assessed. The Mallet Classification System, TAMS, and Box and Block Test were used to evaluate upper limb functional performance [17,29,32,33].

All included studies reported improvements in motor and functional outcomes favouring electrical stimulation-based therapies over controls; however, these findings should be interpreted with caution, given the overall low to very low certainty of the evidence.

### 3.6. Outcome-Specific Findings

This section provides a thorough overview of the effects of NMES and related therapies in children with BPBI by summarizing the findings of the included studies according to particular outcome areas, such as range of motion, muscular strength, and functional performance.

#### 3.6.1. Range of Motion and Joint Function

Five studies assessed joint function and range of motion in children with BPBI. Okafor et al. [10] reported significantly greater improvements in shoulder abduction, elbow flexion, and wrist extension in the electrical stimulation group compared to conventional physiotherapy (shoulder abduction: 8.75° → 36.9° vs. 6.88° → 20°). Sherief [29] reported significant improvements in shoulder flexion, abduction, and external rotation using TAMS (*p* = 0.001). Abdelaziz et al. [30] and Elnegamy [32] demonstrated substantial reductions in biceps and triceps degeneration percentages alongside improved joint mobility (*p* < 0.001). Justice et al. [31] reported a significant improvement in elbow flexion AROM in the first month of NMES (31° vs. −3°, *p* = 0.047), although this effect was not sustained at two and three months. Elnaggar et al. [17] reported significant improvements in shoulder abduction and external rotation according to the Mallet grading system (*p* < 0.05).

While the direction of effect consistently favoured NMES across studies, several methodological limitations substantially constrain interpretation. First, the targeted muscle groups and joints differed across studies. Okafor et al. [10] targeted the shoulder, elbow, and wrist simultaneously; Justice et al. [31] focused exclusively on the biceps and elbow; Elnaggar et al. [17] assessed shoulder function, only making cross-study comparisons clinically inappropriate. Second, four of the five studies [10,17,29,30] did not employ assessor blinding, introducing a substantial risk of detection bias that may have inflated observed gains, particularly for goniometric measurements. Third, none of the studies reported confidence intervals for between-group differences, and most provided *p*-values only [10,29,30,32], precluding any assessment of the precision or clinical magnitude of the reported effects. Fourth, the significant improvement observed by Justice et al. [31] at one month was not maintained at later time points, raising questions about the durability of NMES-related gains in joint mobility. Taken together, while a potential positive effect of NMES on the range of motion cannot be excluded, the current evidence is of very low certainty and insufficient to support definitive conclusions.

#### 3.6.2. Muscle Strength and Motor Function

All seven included studies assessed muscle strength and motor function outcomes [10,17,29,30,31,32,33]. Okafor et al. [10] reported greater mid-arm circumference gains in the electrical stimulation group (14.63 → 16.56 cm) alongside superior upper limb function compared to controls. Sherief [29] reported significant reductions in deltoid (68.8 → 41.9%) and biceps (69.6 → 53.2%) degeneration percentages combined with improved TAMS scores (*p* = 0.001). Abdelaziz et al. [30] and Elnegamy [32] reported significantly greater reductions in biceps and triceps degeneration ratios in the NMES groups (*p* < 0.001). Justice et al. [31] confirmed early motor gains in elbow flexion following NMES, with biceps muscle strength improving from MRC grade 2 to 3 over three months. Elnaggar et al. [17] demonstrated that NMES during weight-bearing exercises produced significant improvements in Mallet score components compared to physiotherapy alone (*p* < 0.05). Tariq et al. [33] found that the combination of NMES and CIMT produced greater augmentation in EMG activation, co-contraction ratio, and root mean square values of wrist extensors compared to NMES alone (*p* < 0.05).

Despite the apparent consistency in the direction of findings, several critical issues limit confidence in these results. The outcome measures used to assess muscle strength were highly heterogeneous across studies [10,17,29,30,31,32,33]: studies employed TAMS, ENoG degeneration percentages, mid-arm circumference, MRC scale, and surface EMG, none of which are directly comparable. ENoG degeneration percentage is a surrogate electrophysiological measure whose relationship to clinically meaningful functional recovery has not been established in this population [30,32], limiting the interpretability of improvements reported by Sherief [29], Abdelaziz et al. [30], and Elnegamy [32]. Furthermore, the absence of assessor blinding in five of seven studies [10,17,29,30,33] is particularly consequential for subjective and semi-objective strength assessments, where observer expectation can substantially influence scoring. The claim that NMES reduces muscle atrophy and promotes neuromuscular activation, while biologically plausible [34,35], is not directly demonstrated by the available evidence; most studies infer these mechanisms from indirect measures rather than histological or neurophysiological confirmation. Overall, the certainty of evidence for muscle strength and motor function outcomes is low to very low, and findings should be regarded as preliminary.

#### 3.6.3. Functional Tests

Five studies used standardised outcome measures to evaluate functional performance [17,29,31,32,33]. Sherief [29] reported significant improvements in TAMS ratings for shoulder flexion (2.6 → 5.4), abduction (3.3 → 6.6), and external rotation (2.6 → 6.8) in the electrical stimulation group (*p* = 0.001). Elnaggar et al. [17] demonstrated that the NMES group outperformed controls across all Mallet score components, including hand-to-mouth, hand-to-back, and hand-behind-head movements (*p* < 0.05). Elnegamy [32] reported significant improvements in TAMS for both biceps and triceps functions following reciprocal electrical stimulation (*p* < 0.001). Justice et al. [31] confirmed early functional gains in elbow flexion during the first month of NMES, alongside a reduction in biceps substitution patterns and no reported adverse effects. In a recently published RCT, Tariq et al. [33] found that the CIMT + NMES group achieved significantly higher Box and Block Test scores (9.64 → 130.55 blocks, *p* < 0.001) and Mallet scores (1.00 → 4.06, *p* < 0.001) within the group over time; however, the between-group comparison with the NMES-only group did not reach statistical significance for either outcome (Mallet: *p* = 0.101; Box and Block: *p* = 0.054).

A critical appraisal of these findings reveals important limitations that temper their interpretation. The functional outcome measures used across studies, TAMS [29,32], Mallet score [17,33], and Box and Block Test [33], assess different constructs and different age groups and are not interchangeable. TAMS is validated for infants and young children, the Mallet score for older children, and the Box and Block Test primarily reflects gross manual dexterity; synthesizing across these instruments therefore risks conflating distinct functional domains. More importantly, the only study reporting a direct between-group comparison on a task-based functional test that included an NMES-only comparator Tariq et al. [33] failed to demonstrate a statistically significant advantage for the combined intervention, despite substantial within-group gains. This suggests that the large improvements observed within NMES groups across studies [17,29,32] may partly reflect natural recovery trajectories or regression to the mean rather than a true treatment effect, a possibility that cannot be excluded given the absence of untreated control groups in most studies. Additionally, assessor blinding was absent in three of the five studies assessing functional outcomes [17,29,33], further increasing the risk that observed gains were influenced by observer expectation. The certainty of evidence for functional performance outcomes is therefore very low, and the apparent benefits of NMES on standardized functional tests require confirmation from adequately powered, methodologically rigorous trials.

### 3.7. Certainty of Evidence (GRADE Assessment)

The certainty of evidence for each outcome was assessed using the GRADE approach (Table 3). Overall, the certainty of evidence ranged from low to very low across all outcomes. Evidence related to range of motion, muscle strength, and motor function was rated as low certainty due to serious risk of bias, inconsistency across studies, and imprecision associated with small sample sizes. Outcomes related to electrophysiological measures and functional performance were rated as very low certainty, primarily due to the limited number of studies and greater heterogeneity. No outcome reached a moderate or high level of certainty.

## 4. Discussion

This systematic review looked at how neuromuscular electrical stimulation (NMES) affected infants or children with brachial plexus birth injury (BPBI) in terms of muscle strength, range of motion, motor function, and upper extremity performance. The results of 7 randomized controlled trials [10,17,29,30,31,32,33] served as the basis for the conclusions. The findings of this systematic review suggest that NMES may contribute to improvements in motor activation, muscle strength, and upper limb function in children with BPBI; however, these findings should be interpreted with caution, given the overall low certainty of the evidence and methodological limitations of the included studies.

Significant improvements in upper extremity actions, including wrist extension, elbow flexion, and shoulder abduction, were found in more than half of the reviewed trials [10,17,29,30,31,32]. Specifically, shoulder abduction and external rotation improved more quickly and significantly in the NMES groups than in the control groups, according to Okafor et al. [10] and Elnaggar et al. [17]. These results suggest that electrical stimulation may promote the reactivation of motor units when combined with both passive and dynamic activities. Furthermore, Sherief [29] and Abdelaziz et al. [30] showed that NMES improved electrical conductivity and decreased the rate of muscle fiber degradation, particularly in the deltoid and biceps muscles. Although these findings generally favored NMES, the certainty of the evidence for these outcomes was rated as low to very low because of methodological limitations, high risk of bias, small sample sizes, and substantial heterogeneity across studies. Therefore, the observed improvements should be interpreted cautiously, and the true effect of NMES may differ from the currently reported findings.

Five of the studies reviewed [17,29,31,32,33] demonstrated significant improvements in functional performance. NMES groups showed greater improvement than control groups according to standardized outcome measures such as the Box and Block Test, TAMS, and Mallet Score. These findings suggest that NMES may be associated with improvements in muscle strength and functional tasks (such as hand-mouth, hand-neck, and hand-back movements) performance; however, the certainty of this evidence is low. In addition, Justice et al. [31] reported that NMES was safe and may improve active elbow flexion in BPBI during the first month, with increased biceps muscle strength and reduced substitution patterns, without any reported adverse effects. Although these findings generally favored NMES, the certainty of evidence was low to very low, and the underlying mechanisms remain uncertain.

The included studies demonstrated moderate methodological quality, with PEDro scores ranging from 5 to 8 (mean = 5.7). However, participant and therapist blinding were reported in only one study [31], which may increase the risk of performance bias in the remaining studies. More importantly, the generalizability of the results was limited by substantial heterogeneity in NMES procedures across studies. Differences in frequency, pulse duration, waveform type, session length, total treatment duration, and targeted muscle groups were evident throughout the literature. For instance, Justice utilized a pulse duration of 300 μs [31], while Abdelaziz and Elnegamy employed 1000 μs [30,32]. Such variability can influence neuronal excitability and muscle fiber activation, potentially contributing to inconsistent outcomes. Although the ideal NMES frequency and duration remain a topic of debate, current research suggests that early, regular, and targeted NMES may enhance functional gains and support nerve regeneration [36].

A more detailed study-level evaluation reveals important methodological weaknesses that may have influenced the reported outcomes. For instance, Okafor et al. [10], Sherief [29], Abdelaziz et al. [30], and Tariq et al. [33], which had lower PEDro scores (5/10), did not report allocation concealment or assessor blinding, increasing the risk of selection and detection bias. These limitations may have systematically inflated the observed treatment effects in favour of NMES assessor; blinding absence increases the risk of detection bias in goniometric and functional scale measurements, while the lack of participant and therapist blinding may have introduced performance bias through differential motivation and attention between groups. Furthermore, the absence of allocation concealment increases susceptibility to selection bias, and consistently small sample sizes amplify the risk of chance findings. Collectively, these factors suggest that the true effect of NMES may be more modest than currently reported.

In contrast, Justice et al. [31], which achieved the highest methodological quality score (PEDro 8/10), incorporated participant, therapist, and assessor blinding. Elnegamy et al. [32] also included assessor blinding but demonstrated only moderate methodological quality overall (PEDro 6/10). Nevertheless, methodological limitations remained across the evidence base, particularly regarding blinding procedures. No study reported intention-to-treat analysis, which may increase the risk of attrition bias and limit confidence in the reported treatment effects. This is especially important given the relatively small sample sizes, where participant loss could substantially influence the results. Importantly, although several studies reported positive effects of NMES, the certainty of the evidence according to the GRADE assessment ranged from low to very low for most outcomes. The certainty of the evidence was downgraded primarily because of methodological limitations, small sample sizes, imprecision, and substantial clinical heterogeneity across studies. Therefore, the findings of this review should be interpreted cautiously, and strong clinical recommendations cannot currently be made.

The findings of this review suggest that NMES may contribute to functional improvement; however, current evidence is of low certainty and does not allow definitive clinical recommendations. Although the clinical studies included in this review did not directly measure neuroplasticity, previous research suggests that NMES can modulate corticospinal activity and synaptic reorganization [34]. Children’s strong neural plasticity potential may make these effects more pronounced [35]. Therefore, NMES may have the potential to improve short- and long-term functional outcomes when combined with conventional physical and occupational therapy approaches. Taken together, these findings suggest that NMES-based therapies may have clinical relevance; however, current evidence remains insufficient to draw firm conclusions regarding their effectiveness.

From a clinical perspective, NMES is a practical, non-invasive, and generally safe intervention when used appropriately for pediatric rehabilitation [36]. However, these considerations should be interpreted cautiously, as the certainty of the evidence was rated as low to very low across most outcomes. Although optimal stimulation parameters have not been established, studies reporting positive findings commonly used frequencies between 20 and 35 Hz and pulse durations ranging from 300 to 1000 μs. Current evidence supports the use of NMES as an adjunct to conventional physical and occupational therapy rather than as a standalone intervention. Stimulation intensity should be individualized to achieve a visible muscle contraction while maintaining patient comfort. Particular attention should be given to patient age, injury severity, skin integrity, and treatment tolerance. In home-based applications, caregiver education and regular clinical monitoring are recommended to ensure safe and appropriate use. Its compatibility with conventional therapy and potential for home-based application, particularly when administered by parents, highlights its accessibility and cost-effectiveness. However, despite these advantages, several important barriers may limit its widespread implementation. Effective use of NMES requires adequate parental training, appropriate device accessibility, and careful monitoring to ensure correct application and safety. In addition, device costs and variability in clinical guidance may further restrict its use in certain settings. Therefore, while NMES may have potential clinical applicability, these practical considerations should be considered when interpreting its translation into clinical practice and real-world settings, as well as when designing future rehabilitation protocols.

The muscle groups that NMES targets differ significantly between studies. For example, Justice et al. only targeted the biceps [31], Tariq et al. administered NMES to the wrist extensors [33], and Okafor et al. concentrated on the shoulder abductors, elbow flexors, and wrist extensors [10]. These differences may be due to variations in the degree of nerve innervation, the severity of the lesion, and the function of the targeted muscle, depending on the situation. Therefore, it should be noted that this situation makes it difficult to comment on the generalizability of NMES effects and to make direct comparisons.

Another significant disadvantage is that stimulation parameters are missing or inadequately reported in nearly half of the studies. In the experiments conducted by Okafor, Sherief, and Tariq [10,29,33], important technical parameters such as frequency, current type, power, and pulse duration were not specified, which limited the reproducibility of the interventions.

Additionally, differences were observed in the duration and frequency of NMES application; protocols ranged from 6 to 12 weeks, and sessions were performed three to seven times per week. This variability contributes to differences in overall stimulation dose, thereby increasing the variability of results. Although longer-term and more frequent procedures have been shown to increase muscle activation and improve function [17,30], the generalizability of these results is limited by the fact that there has been only one long-term follow-up study [31], which showed that the effect was not maintained over the long term.

Furthermore, there is a great deal of uncertainty around the clinical application of NMES for children with BPBI, although interest in it is expanding. There are practical issues, such as whether NMES should be administered after direct current (DC) stimulation, and the best time to start in babies is still unknown. Variability in clinical practice is a result of these ambiguities. Additionally, while NMES has been studied in this population, most of the evidence that is currently available comes from case series rather than high-quality randomized controlled trials. Most of these studies come from a similar region as well, which shows the need for more diverse regions and varied research in this field.

### 4.1. Clinical Significance of the Findings

The integration of NMES into rehabilitation programs for children with BPBI can be seen as a method that helps with functional improvement, and this is one of the most important clinical conclusions of the study. NMES may contribute to the restoration of spontaneous movement in some patients; however, evidence supporting this effect remains limited.

It is also very important to remember that traditional rehabilitation practices often include NMES. Looking more carefully at the literature, although this review mainly includes studies that directly compare NMES with other interventions, it is seen that most traditional treatments already include NMES, strengthening exercises, AROM exercises, and, in certain cases, the use of splints [37,38,39,40]. In conclusion, although some studies have applied NMES as a standalone treatment, its complementary role in improving functional outcomes may be beneficial when used alongside other therapies that have been studied and proven effective.

Clinically, NMES can be included in home-based programs due to its non-invasive design, ease of application, and compatibility with conventional therapy. However, when developing a treatment plan, the patient’s age, degree of injury, muscle sensitivity, and tolerance must be considered. If NMES is applied incorrectly, patients may experience discomfort, or muscles may not be sufficiently activated. Therefore, this intervention must be administered by qualified physical therapists in a personalized manner.

Although neuroplasticity was not directly assessed in the included studies, recent evidence suggests that NMES may promote activity-dependent neural plasticity through both peripheral and central mechanisms. Electrical stimulation has been associated with increased expression of neurotrophic factors involved in nerve regeneration, as well as enhanced corticospinal excitability and sensorimotor reorganization, which may support motor recovery and functional improvement [40,41]. Taken together, these neurophysiological mechanisms suggest that NMES may facilitate peripheral nerve regeneration and muscle reinnervation through both neurotrophic and activity-dependent pathways. In the context of BPBI, where denervation, impaired motor unit activation, and subsequent muscle atrophy are key pathological features, these mechanisms provide a biological rationale for the potential role of NMES in supporting functional recovery [40].

From a clinical perspective, NMES may be particularly beneficial in children with reduced voluntary muscle activation and early-stage motor impairment. Clinicians are advised to apply NMES in combination with active rehabilitation approaches rather than as a standalone intervention. Stimulation intensity should be individualized to elicit visible muscle contraction without causing discomfort. In addition, careful consideration should be given to patient age, injury severity, and tolerance when designing treatment protocols. Close monitoring is especially important in home-based applications to ensure safe and effective use.

### 4.2. Strengths and Limitations

A major strength of this review is the inclusion of only randomized controlled trials, together with the systematic assessment of risk of bias using RoB 2 and certainty of evidence using the GRADE approach. Furthermore, the review incorporates the most recent available evidence, including studies published after the review by Justice et al. [12]. However, several limitations should be acknowledged. Most of the included studies had small sample sizes and heterogeneous participant characteristics. Significant differences were observed in NMES protocols with respect to frequency, duration, intensity, stimulation parameters, and electrode placement, which limited the comparability of the results. In addition, there was substantial variability in participant characteristics, as studies included a wide age range from infancy to early childhood, which further contributed to clinical heterogeneity. Age may influence spontaneous recovery potential, neuromuscular maturation, cortical plasticity, treatment tolerance, and responsiveness to NMES. Therefore, treatment effects observed in infants may not be directly comparable to those observed in older children. Although age-based subgroup analyses would have been clinically meaningful, studies within similar age categories differed substantially in target muscle groups, intervention protocols, and outcome measures, making meaningful subgroup comparisons unreliable. Furthermore, some studies had limited follow-up periods and methodological limitations, including inadequate blinding procedures, making it difficult to draw definitive conclusions regarding long-term effectiveness. Spontaneous recovery during infancy may have contributed to the observed improvements and confounded the independent effects of NMES. Another limitation was that standardized effect sizes could not be calculated consistently across studies because of incomplete reporting and substantial heterogeneity in outcome measures and data presentation. Consequently, pooled SMD (standardized mean difference) analyses, which could have quantitatively supported the clinical significance of the findings, could not be performed. In addition, publication bias cannot be completely excluded, as studies with small sample sizes or negative findings are less likely to be published. Therefore, although this review provides a comprehensive synthesis of the current evidence, the findings should be interpreted cautiously due to the limited number of studies, substantial clinical heterogeneity, and the overall low to very low certainty of the evidence.

Future research should focus on adequately powered randomized controlled trials using standardized NMES protocols, validated and age-appropriate outcome measures such as TAMS for infants, Mallet score for older children, goniometric AROM, and MRC scale for muscle strength, and longer follow-up periods. In addition, studies investigating age-specific treatment approaches, particularly the potential benefits of earlier intervention, optimal stimulation parameters, dose–response relationships, and neurophysiological correlates of recovery are needed to strengthen the evidence base for NMES in children with BPBI and to support the development of standardized clinical guidelines.

## 5. Conclusions

Current evidence suggests that NMES may improve range of motion, muscle strength, and functional performance in children with brachial plexus birth injury. However, the certainty of evidence remains low to very low due to methodological limitations, high risk of bias, small sample sizes, and substantial clinical heterogeneity among studies. Therefore, although NMES may represent a promising adjunct to conventional rehabilitation, definitive clinical recommendations cannot currently be made. Future adequately powered randomized controlled trials with standardized protocols, validated outcome measures, and longer follow-up periods are required to establish the effectiveness and optimal application of NMES in this population.

## Figures and Tables

**Table 4:** PEDro Assessment Scores of Included Studies.

PEDro Criteria												
References	Q1 *	Q2	Q3	Q4	Q5	Q6	Q7	Q8	Q9	Q10	Q11	Score
Okafor et al., 2008 [10]	N	Y	N	Y	N	N	N	Y	N	Y	Y	5/10
Sherief, A.A.A., 2011 [29]	Y	Y	N	Y	N	N	N	Y	N	Y	Y	5/10
Elnaggar et al., 2016 [17]	Y	Y	Y	Y	N	N	N	Y	N	Y	Y	6/10
Abdelaziz et al., 2022 [30]	Y	Y	N	Y	N	N	N	Y	N	Y	Y	5/10
Justice et al., 2023 [31]	Y	Y	N	Y	Y	Y	Y	Y	N	Y	Y	8/10
Elnegamy, T.E., 2024 [32]	N	Y	N	Y	N	N	Y	Y	N	Y	Y	6/10
Tariq et al., 2024 [33]	Y	Y	N	Y	N	N	N	Y	N	Y	Y	5/10

Q1: Eligibility criteria specified, Q2: Random allocation, Q3: Allocation concealed, Q4: Baseline comparability, Q5: Blinding of subjects, Q6: Blinding of therapists, Q7: Blinding of assessors, Q8: Adequate follow-up (>85%), Q9: Intention-to-treat analysis, Q10: Between-group differences reported, Q11: Point estimates and variability provided, Abbreviations: Y: Yes, N: No; *: Eligibility criteria do not contribute to the total score.

## Data Availability

The data generated in this study are all presented in the manuscript.

## Supplementary Materials

The following supporting information can be downloaded at: https://www.mdpi.com/article/10.3390/medicina62061143/s1. File S1. Search Strategy; File S2. PRISMA·2020·Checklist [42].

## Abbreviations

The following abbreviations are used in this manuscript:

AROM	Active Range of Motion
BMD	Bone Mineral Density
BPBI	Brachial Plexus Birth Injury
CIMT	Constraint-Induced Movement Therapy
CT	Conventional Therapy/Control
DC	Direct Current
DEXA	Dual-Energy X-ray Absorptiometry
EMG	Electromyography
ENoG	Electroneurography
Hz	Hertz
MRC	Medical Research Council (muscle strength scale)
MSS	Mallet Score System
MST	Mallet Score Test
μs	Microseconds
NMES	Neuromuscular Electrical Stimulation
OBPI	Obstetric Brachial Plexus Injury
OBPP	Obstetric Brachial Plexus Palsy
PICOS	Population, Intervention, Comparison, Outcome, Study Design
pps	Pulses per Second
PRISMA	Preferred Reporting Items for Systematic Reviews and Meta-Analyses
PROSPERO	International Prospective Register of Systematic Reviews
PT	Physical Therapy (Physiotherapy)
RES	Reciprocal Electrical Stimulation
RCT	Randomized Controlled Trial
ROB 2	Risk of Bias 2 Tool
ROM	Range of Motion
s	Seconds
SD	Standard Deviation
