# Supplementary material for: Neuromuscular Electrical Stimulation in Brachial Plexus Birth Injury Rehabilitation: A Systematic Review
Source: Medicina (Kaunas). 2026 Jun 11;62(6):1143. doi: 10.3390/medicina62061143 (PMC13304322; doi:10.3390/medicina62061143)
Supplement: Supplementary file 1 [file medicina-62-01143-s001.zip › File S1. Search Strategy.pdf]

Appendix 1: Search Strategy

All Search Database Results and Duplicate Results

**Search Database**	Results
Pubmed	305
Scopus	149
Web of Science	14
PEDro	50
CINAHL	865
Cochrane	50
Total	1433
Duplicates	684
Unique	749

Pubmed

Search number	Query	Results
4	#1 AND #2 AND #3	305
3	"Muscle Strength"[mh] OR "Range of Motion, Articular"[mh] OR "Motor Activity"[mh] OR "motor recovery"[tw] OR "muscle strength"[tw] OR "active range of motion"[tw] OR "upper limb function"[tw] OR "functional recovery"[tw] OR "rehabilitation"[tw]	915,646
2	"electric stimulation therapy"[mh] OR "electric stimulation"[mh] OR "neuromuscular electrical stimulation"[tw] OR " NMES "[tw] OR "electrical stimulation"[tw]	253,235
1	"brachial plexus"[mh] OR "brachial plexus neuropathies "[mh] OR " obstetric brachial plexus "[tw] OR " Erb palsy "[tw] OR " brachial plexus injury "[tw] OR "klumpke palsy "[tw] OR " neonatal brachial plexus palsy"[tw]	31,734

Scopus

Search	Search Terms	Results
S4	S1 AND S2 AND S3	149

S3	TITLE-ABS-KEY ("Muscle Strength") OR TITLE-ABS-KEY ("Range of Motion, Articular") OR TITLE-ABS-KEY (" Motor Activity") TITLE-ABS-KEY ("motor recovery") OR TITLE-ABS-KEY ("active range of motion") TITLE-ABS-KEY ("upper limb function") OR TITLE-ABS-KEY ("functional recovery") OR TITLE-ABS-KEY ("rehabilitation")	832,297
S2	TITLE-ABS-KEY ("electric stimulation therapy") OR TITLE-ABS-KEY ("electric stimulation") OR TITLE-ABS-KEY ("NMES") OR TITLE-ABS-KEY ("neuromuscular electrical stimulation")	133,356
S1	TITLE-ABS-KEY ("brachial plexus") OR TITLE-ABS-KEY ("brachial plexus neuropathies") OR TITLE-ABS-KEY ("obstetric brachial plexus") OR TITLE-ABS-KEY ("Erb palsy") OR TITLE-ABS-KEY ("brachial plexus injury") OR TITLE-ABS-KEY ("brachial plexus injury") OR TITLE-ABS-KEY ("klumpke palsy") OR TITLE-ABS-KEY ("neonatal brachial plexus palsy")	27,701

Web of Science

#	Search Query	Database	Results
1	ALL=("brachial plexus") OR ALL=("brachial plexus neuropathies") OR ALL=("obstetric brachial plexus") OR ALL=("Erb palsy") OR ALL=("brachial plexus injury") OR ALL=("klumpke palsy") OR ALL=("neonatal brachial plexus palsy")	Web of Science Core Collection	16,354
2	ALL=("electric stimulation therapy") OR ALL=("electric stimulation") OR ALL=("neuromuscular electrical stimulation") OR ALL=("NMES") OR ALL=("electrotherapy")	Web of Science Core Collection	11,634
3	ALL=("Muscle Strength") OR ALL=("Range of Motion, Articular") OR ALL=("Motor Activity") OR ALL=("Motor Recovery") OR ALL=("upper limb function") OR ALL=("active range of motion") OR ALL=("functional recovery") OR ALL=("rehabilitation")	Web of Science Core Collection	585,848
4	#3 AND #2 AND #1	Web of Science Core Collection	14

Pedro

Search Terms	Filter	Records
Abstract & Title: “brachial plexus”	Subdiscipline: paediatrics	25 records
Abstract & Title: “neuromuscular electrical stimulation"	Subdiscipline: paediatrics	25 records
Total		50 records

CINAHL Ultimate

#	Query	Limiters/Expanders	Last Run Via	Results
S4	S1 AND S2 AND S3	Limiters - Full Text<br>Expanders - Apply equivalent subjects<br>Search modes - Boolean/Phrase	Interface - EBSCOhost Research Databases<br>Search Screen - Basic Search<br>Database - CINAHL Complete	865
S3	TX " electric stimulation therapy" OR TX "electric stimulation" OR TX "neuromuscular electrical stimulation" OR TX "NMES"	Limiters - Full Text<br>Expanders - Apply equivalent subjects<br>Search modes - Boolean/Phrase	Interface - EBSCOhost Research Databases<br>Search Screen - Basic Search<br>Database - CINAHL Complete	143,063
S2	TX "Muscle Strength" OR TX "motor recovery" OR TX "Range of Motion, Articular" OR TX "upper limb function" OR TX "functional recovery" OR TX "rehabilitation" OR TX "Motor Activity" OR TX "active range of motion" OR TX "Motor Activity"	Limiters - Full Text<br>Expanders - Apply equivalent subjects<br>Search modes - Boolean/Phrase	Interface - EBSCOhost Research Databases<br>Search Screen - Basic Search<br>Database - CINAHL Complete	5,868,024

S1	TX "brachial plexus" OR TX "birth brachial plexus" OR TX "brachial plexus injury" OR TX " Erb palsy" OR TX "klumpke palsy" OR TX "neonatal brachial plexus palsy" OR TX "obstetric brachial plexus" OR TX "brachial plexus neuropathies"	Limiters - Full Text<br>Expanders - Apply equivalent subjects<br>Search modes - Boolean/Phrase	Interface - EBSCOhost Research Databases<br>Search Screen - Basic Search<br>Database - CINAHL Complete	103,823

Cochrane

ID	Search	Hits
#1	[mh "electric stimulation therapy"] OR [mh "electric stimulation"]	14890
#2	[mh “brachial plexus neuropathies”] OR [mh “brachial plexus”]	1320
#3	[mh "Muscle Strength"] OR [mh "Range of Motion, Articular"] OR [mh "Motor Activity"]	57789
#4	("NMES"):ti,ab,kw OR ("neuromuscular electrical stimulation"):ti,ab,kw OR ("electrotherapy"):ti,ab,kw OR ("electric stimulation"):ti,ab,kw	8185
#5	("brachial plexus"):ti,ab,kw OR ("birth brachial plexus"):ti,ab,kw OR ("brachial plexus neuropathies"):ti,ab,kw OR ("obstetric brachial plexus"):ti,ab,kw OR ("Erb palsy"):ti,ab,kw OR ("brachial plexus injury"):ti,ab,kw OR ("klumpke palsy"):ti,ab,kw OR ("neonatal brachial plexus palsy"):ti,ab,kw	3934
#6	("motor recovery"):ti,ab,kw OR ("active range of motion"):ti,ab,kw OR ("Muscle Strength"):ti,ab,kw OR ("upper limb function"):ti,ab,kw OR ("functional recovery"):ti,ab,kw OR ("Motor Activity"):ti,ab,kw OR ("rehabilitation"):ti,ab,kw	112979
#7	#1 or #4	17910
#8	#2 or #5	4578
#9	#3 or #6	149205
#10	#7 AND #8 AND #9	50
